# Automated hippocampal segmentation in 3D MRI using random undersampling with boosting algorithm

**DOI:** 10.1007/s10044-015-0492-0

**Published:** 2015-07-09

**Authors:** Rosalia Maglietta, Nicola Amoroso, Marina Boccardi, Stefania Bruno, Andrea Chincarini, Giovanni B. Frisoni, Paolo Inglese, Alberto Redolfi, Sabina Tangaro, Andrea Tateo, Roberto Bellotti, The Alzheimers Disease Neuroimaging Initiative

**Affiliations:** Istituto di Studi sui Sistemi Intelligenti per l’Automazione, Consiglio Nazionale delle Ricerche, Via G. Amendola 122, 70126 Bari, Italy; Dipartimento Interateneo di Fisica M.Merlin, Universita’ degli Studi di Bari, Bari, Italy; Istituto Nazionale di Fisica Nucleare, Sezione di Bari, Bari, Italy; LENITEM Laboratory of Epidemiology, Neuroimaging and Telemedicine, IRCCS S.Giovanni di Dio, FBF, Brescia, Italy; Overdale Hospital, Saint Helier, Jersey; Istituto Nazionale di Fisica Nucleare, Sezione di Genova, Genova, Italy; AFaR Associazione FateBeneFratelli per la Ricerca, Roma, Italy; Psychogeriatric Ward, IRCCS S.Giovanni di Dio, FBF, Brescia, Italy

**Keywords:** Supervised learning, Classification, Segmentation, MRI

## Abstract

The automated identification of brain structure in Magnetic Resonance Imaging is very important both in neuroscience research and as a possible clinical diagnostic tool. In this study, a novel strategy for fully automated hippocampal segmentation in MRI is presented. It is based on a supervised algorithm, called RUSBoost, which combines data random undersampling with a boosting algorithm. RUSBoost is an algorithm specifically designed for imbalanced classification, suitable for large data sets because it uses random undersampling of the majority class. The RUSBoost performances were compared with those of ADABoost, Random Forest and the publicly available brain segmentation package, FreeSurfer. This study was conducted on a data set of 50 T1-weighted structural brain images. The RUSBoost-based segmentation tool achieved the best results with a Dice’s index of $0.88 \pm 0.01$ ($0.87 \pm 0.01$) for the left (right) brain hemisphere. An independent data set of 50 T1-weighted structural brain scans was used for an independent validation of the fully trained strategies. Again the RUSBoost segmentations compared favorably with manual segmentations with the highest performances among the four tools. Moreover, the Pearson correlation coefficient between hippocampal volumes computed by manual and RUSBoost segmentations was 0.83 (0.82) for left (right) side, statistically significant, and higher than those computed by Adaboost, Random Forest and FreeSurfer. The proposed method may be suitable for accurate, robust and statistically significant segmentations of hippocampi.

## Introduction

The role of neuroimaging in the study of brain disease and for clinical diagnostic purposes has acquired increasing importance. The possibility of investigating the morphology of specific brain structures relies on their accurate delimitation from the surrounding brain parenchyma and from the other adjacent structures (segmentation). This proves particularly challenging for structures characterized by morphological complexity, such as the hippocampus, a part of the temporal lobe with a prominent role in memory and other cognitive functions. The hippocampus is primarily involved in the pathogenesis of a number of conditions, firstly Alzheimer’s disease (AD), the most common type of dementia [1]. Nowadays, a definite diagnosis of AD can only be made if there is histopathological confirmation, either post-mortem or on brain biopsy. However, biomarkers of the disease supportive of the diagnosis are now recognized, and these include structural brain changes visible on Magnetic Resonance Images (MRIs), in particular atrophy of the medial temporal lobe and in particular of the hippocampal formation [2–7].

Manual segmentation of hippocampus has been so far considered the gold standard, despite the heterogeneity of anatomical landmarks and protocols adopted [8]; it is also laborious, time consuming and prone to rater error. Automated segmentation techniques are gaining increasing recognition since, not only they offer the possibility of studying rapidly large databases, for example in pharmaceutical trials or genetic research, but also afford higher test–retest reliability and the robust reproducibility needed for multi-centric studies. In the last few years, state-of-the-art hippocampal segmentation from 3D MRI research has delineated a few major approaches. Multi-atlas methods, among which the joint label fusion technique proposed by Wang et al. [9], are based on information propagation between multiple atlases, and bias correction. Other approaches are based on the active contour models (ACM) [10], in which a deformable contour is iteratively adapted to the image in order to generate the partition of the ROI. Machine learning approaches, on the contrary, use statistical tools from image processing techniques to perform the segmentation of the hippocampus, by focusing on the delineation of most characterizing features (texture, shape, edges). Among them, Morra et al. [11, 12] showed the validity of this approach for accurate segmentation of the hippocampal region. Hence, building accurate tools for the identification of brain structures in MRI is a promising approach to identify anatomical differences that can be associated with the presence or absence of neurodegenerative diseases, such as AD. The brain images mostly contain noise, inhomogeneity and sometime deviation [13], therefore accurate segmentation of brain images in a difficult task. Despite numerous efforts described in the literature [11, 14–19], segmentation is still commonly performed manually by experts.

The main goal of this work was to develop an accurate strategy based on supervised learning algorithms for hippocampal segmentation using 3D brain MRI. The task of a classifier, trained on a set of previously labeled examples (MR images in which the hippocampi had been previously manually segmented), is to classify voxels of a new brain MR image as belonging or not to the hippocampus. In this study, the performance of a novel statistical strategy, based on RUSBoost [20], was evaluated for hippocampal segmentation. RUSBoost was designed for imbalanced classification problems, combining data random undersampling with boosting. It is an alternative of another data sampling/boosting algorithm called SMOTEBoost [21] which uses an oversampling technique, creating new minority class examples by extrapolating between existing examples, combined with boosting technique. Creating new examples, SMOTEBoost increases model training times. It has been successful in applications [22, 23] where not too big data sets were analyzed. As the training data increases in size, the SMOTE run time increases, incurring the risk of becoming impractical. When a data set is very large, as for 3D MRI data sets, selecting an appropriate sampling method becomes important. Training a model on very large data set would take much less if undersampling is used as for RUSBoost. The drawback associated with undersampling is the loss of information that comes with deleting examples from the training data. Moreover, there is evidence that the RUSBoost algorithm performs favorably when compared to SMOTEBoost, while being a simpler and faster technique that often results in significantly better classification performance [21, 24]. To the best of our knowledge, this is the first application of RUSBoost classifiers to hippocampal segmentation.

This work utilizes two datasets, obtained from the Alzheimers Disease Neuroimaging Initiative (ADNI, http://adni.loni.usc.edu/) database, consisting of MR images and their corresponding expert manual labels produced with a standard harmonized protocol. The first data set, DB1, was used for training the algorithms and estimating evaluation metrics via cross validation. The RUSBoost performances on DB1 were excellent when compared with those of three classifiers, Adaboost [25], Random forest (RF) [26] and FreeSurfer v.5.1 [15]. Adaboost is a boosting algorithm that sequentially selects weak classifiers and weights each of them based on their error. It has been previously employed as segmentation tools in [11]. RF uses multiple binary decision trees, and recently several brain MRI segmentation systems based on RF classifiers have appeared in the literature [16, 19, 27–29]. FreeSurfer is a publicly available package and can be considered the state-of-the-art whole-brain segmentation tool, since numerous imaging studies across multiple centers have shown its robustness and accuracy [30].

The second data set, DB2, was employed for an assessment of the performance of the fully trained classifiers. Results on the DB2 data set confirmed those obtained in the previous analysis and showed that the RUSBoost segmentation strategy, trained on DB1, generalized very well on the independent data set, avoiding problems like overfitting. Moreover, the hippocampal volumes obtained with our RUSBoost segmentation showed the best correlation with those segmented manually, which is very important for diagnostic purposes.

For all the classifiers, we also evaluated how the Dice’s index varied with the training set size, providing practical guidelines for future users.

## Materials and methods

### Data set description

The data used in the preparation of this study were obtained from the Alzheimers Disease Neuroimaging Initiative (ADNI, http://adni.loni.usc.edu) database. The ADNI was launched in 2003 by the NIA, the National Institute of Biomedical Imaging and Bioengineering (NIBIB), the U.S. Food and Drug Administration (FDA), private pharmaceutical companies, and nonprofit organizations. For up-to-date information, see http://www.adni-info.org.

Two databases of T1-weighted whole-brain MR images, DB1 and DB2, were used in the study, both including normal controls (NC), subjects with mild cognitive impairment (MCI) and patients with Alzheimer’s disease (AD). All images were downloaded from the ADNI LONI Image Data Archive (https://ida.loni.usc.edu). Both DB1 and DB2 data sets consisted of 50 subjects each whose demographic details are reported in Table 1. All the images were acquired on 1.5 Tesla, and 3.0 Tesla scanners which specifications are reported in Table 2.

**Table 1:** Demographic information of DB1 and DB2 subjects

Data set	Size	Age	Subjects	Number of features
DB1	50	60–89	14 NC, 17 MCI, 19 AD	315
DB2	50	61–90	15 NC, 17 MCI, 18 AD	315

Number of features used in the data sets is shown

**Table 2:** Technical specifications of scanners used to acquire subjects MR images

Manufacturer	Field strength (T)	Acquisition matrix	Slice thickness (mm)	TR (ms)	TE (ms)
Philips medical systems	1.5	$256\times 256\times 170$	1.2	7	3
Philips medical systems	3.0	$256\times 256\times 170$	1.2	7	3
GE medical systems	3.0	$256\times 256\times 166$	1.2	7	3
SIEMENS	1.5	$192\times 192\times 160$	1.2	2300	3
SIEMENS	3.0	$240\times 256\times 160$	1.2	2400	3

*T* tesla (magnet field strength), *TR* repetition time, *TE* echo time

Bilateral hippocampi were manually segmented using the Harmonized Hippocampal Protocol (http://www.hippocampal-protocol.net/) [31, 32] which aims to standardize the available manual segmentation protocols. The more inclusive definition of the Harmonized protocol may also limit the inconsistencies due to the use of arbitrary lines and tissue exclusion of the currently available manual segmentation protocols.

Preprocessing involved a first registration through a six-parameter affine transformation to the Montreal Neurological Institute MNI152 template. Then a gross peri-hippocampal volume was extracted for left and right hippocampi for each scan and for the template; these regions underwent a further affine registration using the template hippocampal boxes as reference images. In this way, two Volumes of Interest (VOIs) of dimension $50 \times 60 \times 60$ were obtained. The two registrations and box extraction were fully automated.

### Features

The 3D segmentation was performed using for each voxel a vector of 315 elements (Table 1) representing information about position, intensity, neighboring texture, and local filters. Haar-like and Haralick features provide information on image texture, in particular on contrast, uniformity, rugosity, regularity, etc. [33–36]. A number of 248 Haar-like features were calculated spanning a 3D filter of varying dimensions (from $3 \times 3 \times 3$ to $9 \times 9 \times 9$) for each voxel and averaging the voxels intensities in each VOI. Forty-eight Haralick features were calculated; in particular energy, contrast, correlation and inverse difference moment were computed based on the calculation of gray level co-occurrence matrix (GLCM), created on the $n \times n$ voxels (*n* varying from 3 to 9) projection subimages of the volume centered in each voxel. A study on local Haralik features has been previously carried out showing their successful application to hippocampal segmentation [27]. Finally, the gradients calculated in different directions and at different distances, and the relative positions of the voxels (*x*, *y*, *z*) were included as additional features.

### RUSBoost

RUSBoost is a boosting-based sampling algorithm designed to handle class imbalance. It combines Random UnderSampling (RUS) and Adaboost. RUS is a technique that randomly removes examples from the majority class until the desired balance is achieved. Let $x _i$ be a point in the feature space *X* and $y_i$ be a class label in $Y=\{-1,+1\}$. The data set S can be represented by the tuple $( x _i, y_i)$ with $i=1, 2,\ldots , m$. The algorithm assigns to each example the weight $D_1(i)=\frac{1}{m}$ for $i=1, 2,\ldots , m$. Then, in each round $t=1, 2 \ldots , T$, the following steps are performed.

1. A temporary training set $S_t'$ is created with distribution $D_t'$ using random undersampling (RUS). It is applied to remove the majority class examples until the percentage *N* of $S_t'$ belongs to the minority class.
2. A weak learner is called providing it with examples $S_t'$ and their weights $D_t'$.
3. A hypothesis $h_t: X \times Y \rightarrow [0,1]$, which associates to every example $x_i$ the probability to get the correct label $y_i$ or the incorrect label $y_i$, is obtained. If $h_t(x_i, y_i) = 1$ and $h_t(x_i, y : y \ne y_i) = 0$ then $h_t$ has correctly predicted that the $x_i$’s label is $y_i$, not *y*. Similarly, if $h_t(x_i, y_i) = 0$ and $h_t(x_i, y : y \ne y_i) = 1$, $h_t$ has incorrectly predicted that the $x_i$’s labels is *y*.
4. The pseudo-loss for *S* and $D_t$ is calculated:
  $$\begin{aligned} \epsilon _t=\sum _{(i,y):y_i\ne y} D_t(i) (1-h_t(x_i,y_i)+h_t(x_i,y)) \end{aligned}$$
  It is a modified version of Adaboost error function: here an higher cost is assigned to the examples with higher probability of being misclassified by the weak learner.
5. The weight update parameter is calculated:
  $$\begin{aligned} \alpha _t=\frac{\epsilon _t}{1-\epsilon _t} \end{aligned}$$
  For $$\epsilon _t \le \frac{1}{2}$$, $$\alpha _t \le 1$$.
6. Update $$D_t$$:
  $$\begin{aligned} D_{t+1}(i)\,=\, & {} D_t(i)\alpha _t^{\frac{1}{2}(1+h_t(x_i,y_i)\,-\,h_t(x_i,y:y\ne y_i))}\\= & {} \left\{ \begin{array}{ll} D_t(i)\alpha _t &{}\text{for } \text{ correctly } \text{ labeled } \text{ examples } \\ D_t(i) &{}\text{for } \text{ misclassified } \text{ examples } \end{array} \right. \end{aligned}$$
  Higher importance is assigned to the mislabeled examples.
7. Normalize $$D_{t+1}$$: $$D_{t+1}(i)=\frac{D_{t+1}(i)}{\sum _i D_{t+1}(i)}$$

Output the final hypothesis:

1 $$\begin{aligned} {\it{H(x )}}=\begin{array}{c} \text{ argmax }\\ y\in Y \end{array} \sum _{t=1}^T h_t( x ,y) \log \frac{1}{\alpha _t}. \end{aligned}$$

## Results and discussion

All data were analyzed using Matlab (MathWorks, Natick, MA).

**Fig. 1:**
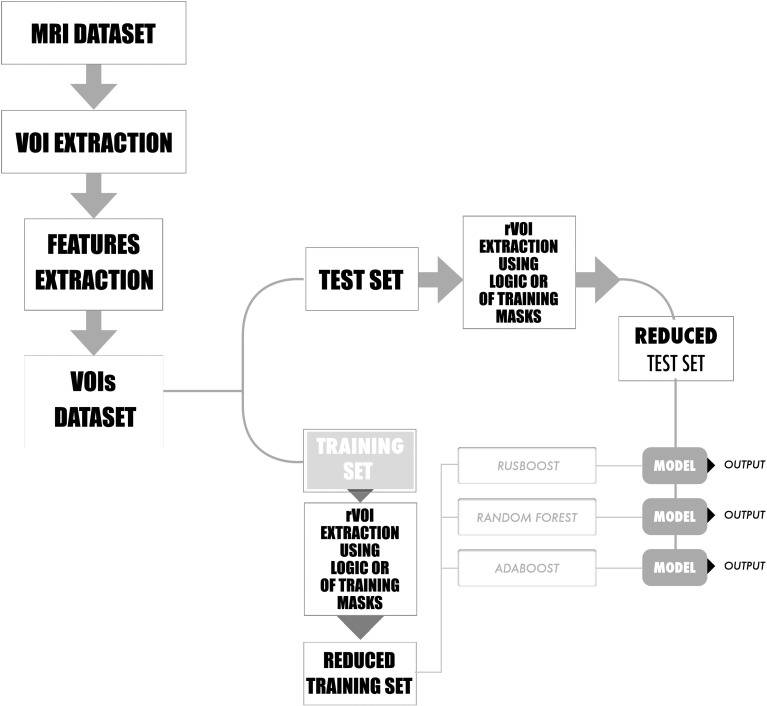
One round of the Cross-Validation technique employed to evaluate the performances of RUSBoost, RF and Adaboost using the data set DB1

A cross-validation (CV) technique was used in order to estimate how accurately a predictive model will perform in practice. Figure 1 shows one round of CV which involves partitioning a sample of data into complementary subsets, training and test sets, building the classifier on the first set, and validating the model on the second set. To reduce variability, multiple rounds of CV are performed using different partitions, and the validation results are averaged over the rounds.

Before performing the classification, the preprocessing involved a first registration of all the images in the same stereotaxic space and extraction of the gross peri-hippocampal VOI containing $50 \times 60 \times 60 = 180000$ voxels (see Sect. 2.1). Next 315 features suitable for describing complex images were extracted, as reported in Sect. 2. Hence the number of examples in the training (test) set was given by 180000 $\times$ the number of training (test) images, and the number of components was 315. Internally to each round of the cross validation, a bounding box around the training hippocampi was defined by the logical OR of the training masks. A reduced VOI (rVOI) was identified using this bounding box plus some neighboring voxels obtained applying a cubic kernel of size $2 \times 2 \times 2$. The rVOI dimensions increased with the number *m* of training images (with $m = 5, 10, 15,\ldots , 40$) and in each round of the CV, the rVOIs changed. The rVOI dimensions over ten rounds of CV were averaged. The resulting mean values, varying *m*, are shown in the Table 3. Reduced training set and test set were built based on the training rVOI; their size can be computed multiplying the rVOI size by the number of training/test images.

**Table 3:** Mean values of rVOI sizes computed over 10 rounds of CV, varying the number *m* of training images for left and right brain hemispheres

*m*	Left	Right
5	13436	14683
10	14772	15261
15	16206	17189
20	17385	18409
25	17830	18760
30	18403	20000
35	18921	22301
40	19282	20642

The voxels outside the training rVOI definitely do not belong to the hippocampus. The neighboring voxels were included because they might contain hippocampal voxels of testing images lying outside the bounding box. The percentage of hippocampal voxels in the training rVOIs was in the range of 27–38 % of the total number. The use of rVOIs also reduced the computational time required for training the classifiers. It is worth reporting that in a first attempt, random undersampling of the majority class was used to obtain a desired unbalancing (in the range of 25–40 %) between hippocampus and non-hippocampus sets, combined with the classification task. This procedure results in worsened performances of the classifiers, hence the rVOI extraction was adopted.

A number of standard metrics, described in Appendix 1, were calculated for each segmentation algorithm: Dice’s coefficient, Precision, Recall and Relative Overlap (R.O). In particular Dice’s index was used to compare the performances of the methods [12]. Left and right hemispheres were independently analyzed.

The RUSBoost performance for automated segmentation on the DB1 MRI data set was studied. The RUSBoost algorithm provided by the fitensemble function in the Statistics Toolbox of Matlab was used. The relationship between the evaluation metrics and the number *m* of training VOIs, with $m=5, 10, 15,\ldots , 40$, was evaluated using the strategy shown in Fig. 1, with 10 CVs. Parameters tuning of RUSBoost was performed on a wide range of values: number of rounds *T* equal to $10, 50, 100, 150, 200, 250,\ldots , 500$ and learning rate equal to $0.01, 0.05, 0.1, 0.2, \ldots , 1.$ The optimal number of rounds was $T=150$, the learning rate equal to 0.1, and the desired percentage of minority class was set at the default value of $N=50\,\%$. The results, illustrated in Table 4, highlighted the excellent performances of RUSBoost which provided a Dice’s index of 0.84 with only 10 training images. Its ability to separate hippocampal from background voxels improved as the number of training VOIs increased. The best performances of RUSBoost were obtained with $m = 30$ training VOIs with a Dice’s coefficient of $0.88 \pm 0.01$ for the left, and $0.87 \pm 0.01$ for the right side. The Dice’s coefficient did not improve by increasing further the number of training VOIs, suggesting that $m = 30$ was the optimal number.

**Table 4:** Dice, precision, recall and relative overlap are reported for RUSBoost analysis on left and right brain hemispheres varying the number *m* of training VOIs

*m*	Dice	Precision	Recall	R.O.
*RUSBoost—left hemisphere*				
5	0.8060 $\pm$ 0.0180	0.8300 $\pm$ 0.0162	0.7878 $\pm$ 0.0321	0.6776 $\pm$ 0.0257
10	0.8402 $\pm$ 0.0084	0.8623 $\pm$ 0.0109	0.8226 $\pm$ 0.0156	0.7264 $\pm$ 0.0122
15	0.8524 $\pm$ 0.0053	0.8677 $\pm$ 0.0102	0.8402 $\pm$ 0.0097	0.7444 $\pm$ 0.0045
20	0.8557 $\pm$ 0.0054	0.8969 $\pm$ 0.0075	0.8444 $\pm$ 0.0049	0.7494 $\pm$ 0.0062
25	0.8610 $\pm$ 0.0058	0.8716 $\pm$ 0.0156	0.8534 $\pm$ 0.0160	0.7573 $\pm$ 0.0072
**30**	**0.8797** $\pm$ **0.0053**	**0.8794** $\pm$ **0.0101**	**0.8675** $\pm$ **0.0137**	**0.7801** $\pm$ **0.0065**
35	0.8773 $\pm$ 0.0100	0.8800 $\pm$ 0.0105	0.8644 $\pm$ 0.0154	0.7800 $\pm$ 0.0138
40	0.8763 $\pm$ 0.0111	0.8840 $\pm$ 0.0121	0.8621 $\pm$ 0.0164	0.7808 $\pm$ 0.0165
*RUSBoost—right hemisphere*				
5	0.8042 $\pm$ 0.0120	0.8277 $\pm$ 0.0248	0.7900 $\pm$ 0.0209	0.6641 $\pm$ 0.0166
10	0.8377 $\pm$ 0.0077	0.8572 $\pm$ 0.0215	0.8250 $\pm$ 0.0217	0.7232 $\pm$ 0.0108
15	0.8501 $\pm$ 0.0060	0.8711 $\pm$ 0.0097	0.8344 $\pm$ 0.0091	0.7419 $\pm$ 0.0082
20	0.8586 $\pm$ 0.0050	0.8726 $\pm$ 0.0166	0.8489 $\pm$ 0.0125	0.7541 $\pm$ 0.0071
25	0.8645 $\pm$ 0.0059	0.8744 $\pm$ 0.0099	0.8594 $\pm$ 0.0151	0.7630 $\pm$ 0.0081
**30**	**0.8676** $\pm$ **0.0092**	**0.8825** $\pm$ **0.0135**	**0.8571** $\pm$ **0.0144**	**0.7680** $\pm$ **0.0133**
35	0.8670 $\pm$ 0.0211	0.8815 $\pm$ 0.0163	0.8502 $\pm$ 0.0199	0.7630 $\pm$ 0.0212
40	0.8669 $\pm$ 0.0220	0.8806 $\pm$ 0.0174	0.8481 $\pm$ 0.0254	0.7618 $\pm$ 0.0322

The analysis was performed using the DB1 data set. Means and standard deviations values, measured over 10 rounds of cross validation, are shown

**Fig. 2:**
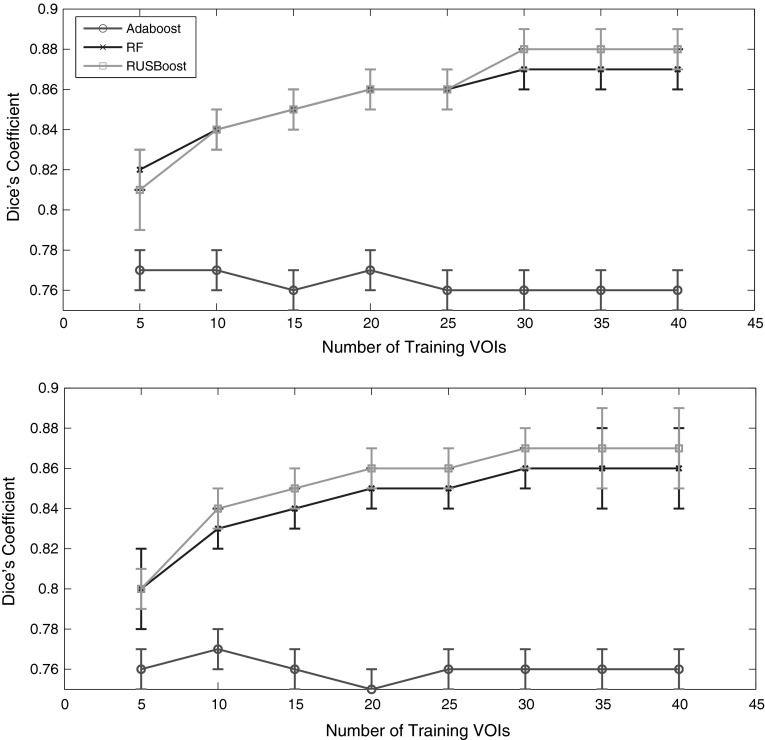
Cross-validation Dice’s coefficients of RUSBoost, Adaboost and RF classifiers varying the number of training brain images on *left* and *right* brain hemispheres, using DB1 data set. *Error bars* represents standard deviations

Subsequently, we compared the performances of RUSBoost with two classifiers previously used in medical image analysis [11, 27, 28]: Adaboost and RF (see Appendix 1). Figure 2 summarizes the relationship between the Dice’s coefficients of the three classifiers and the number of training VOIs. Parameters tuning of Adaboost was performed using a number of rounds *T* equal to $10, 50, 100, 150, 200, 250,\ldots , 500$ and learning rate equal to $0.01, 0.05, 0.1, 0.2, \ldots , 1$; the optimal number of boosting rounds was $T = 400$ and its learning rate 0.1. Parameter tuning of RF was performed using the number of trees equal to $10, 50, 100, 150, 200, 250,\ldots , 500$ and the optimal number resulted to be 150. The metrics values were estimated performing ten cross validations. The best performance of Adaboost on DB1 data set was reached with few training VOIs, providing a Dice’s index of about 0.77. The figure shows that Adaboost had a limited learning ability, because the Dice’s coefficient did not increase significantly as the number of training examples increased, and its performances were very poor compared with those of RUSBoost and RF. The advantage of combining the RUS with boosting appeared conspicuous. As already seen for RUSBoost, the Dice’s coefficients of the RF classifiers increased with the number of training VOIs and the curves leveled off after 30 training images, indicating that it would be pointless increase further the number of images. The best performances of RF were obtained using $m = 30$ VOIs with a dice’s index of $0.87 \pm 0.01$ for left and $0.86 \pm 0.01$ for right hemispheres, in agreement with RUSBoost results. Table 5 shows all the metrics values obtained using $m = 30$ training VOIs for left and right hemispheres, highlighting a strong concordance of results between the two brain hemispheres.

**Table 5:** Dice, precision, recall and relative overlap are reported for RUSBoost, Adaboost, RF and FreeSurfer v.5.1 analysis on left and right brain hemispheres of the DB1 data set

	Dice	Precision	Recall	R.O.
*Left*				
RUSBoost	0.8797 $\pm$ 0.0053	0.8794 $\pm$ 0.0101	0.8675 $\pm$ 0.0137	0.7801 $\pm$ 0.0065
Adaboost	0.7595 $\pm$ 0.0053	0.7671 $\pm$ 0.0077	0.7615 $\pm$ 0.0085	0.6142 $\pm$ 0.0054
RF	0.8675 $\pm$ 0.0055	0.8926 $\pm$ 0.0057	0.8464 $\pm$ 0.0083	0.7670 $\pm$ 0.0070
FreeSurfer	0.7420 $\pm$ 0.0496	0.6880 $\pm$ 0.0680	0.5550 $\pm$ 0.0531	0.7120 $\pm$ 0.0477
*Right*				
RUSBoost	0.8676 $\pm$ 0.0092	0.8825 $\pm$ 0.0135	0.8571 $\pm$ 0.0144	0.7680 $\pm$ 0.0133
Adaboost	0.7595 $\pm$ 0.0060	0.7671 $\pm$ 0.0077	0.7615 $\pm$ 0.0085	0.6142 $\pm$ 0.0054
RF	0.8602 $\pm$ 0.0124	0.9138 $\pm$ 0.0088	0.8154 $\pm$ 0.0188	0.7571 $\pm$ 0.0179
FreeSurfer	0.7560 $\pm$ 0.0451	0.6850 $\pm$ 0.0743	0.5600 $\pm$ 0.0574	0.7160 $\pm$ 0.0526

Means and standard deviations values, measured over 10 rounds of cross validation, with $m=30$ training brain MR images are shown. The RUSBoost numbers are the same reported in Table I and are reproduced here for consistency

RUSBoost showed higher Recall than RF: the $87\,\%$ ($86\,\%$) of true left (right) hippocampus was correctly identified by RUSBoost, versus the $85\,\%$ ($82\,\%$) identified by RF. The Precision with RF was slightly higher than that of RUSBoost: $89\,\%$ ($91\,\%$) of the voxels that RF predicted as hippocampus for the left (right) side, was true hippocampus. This was $88\,\%$ with RUSBoost.

Finally, in Table 5 the RUSBoost behavior was compared the publicly available segmentation package FreeSurfer v.5.1 (see Appendix 1), highlighting the excellent segmentation performances of the proposed algorithm. FreeSurfer segmentations compared with manual segmentations similarly to Adaboost, with a Dice’s coefficient of 0.74 (0.76) for the left (right) side. These numbers should be treated with caution, because FreeSurfers segmentation tool uses a probabilistic atlas constructed from training data different from those employed for other algorithms, and an exact comparison is not possible without using the same data. To overcome this drawback, the performances of all the segmentation methods were evaluated on an independent data set. With this aim, we used an external data set DB2, obtained from an ADNI archive. This procedure guarantees a bias-free estimations of metrics for the RUSBoost, Adaboost and RF final model, trained on DB1, since DB2 was not employed to select the final models. For this section of the study, FreeSurfer was used again for comparison. The results (Table 6) illustrate the excellent performance of RUSBoost, followed by RF, on DB2, in keeping with the DB1 analysis. Unlike the DB1 analysis, in this case RUSBoots also achieved best Precision (0.89 and 0.88 for left and right side) and Recall (0.86 and 0.85 for left and right side). FreeSurfer and Adaboost gave the worst results.

**Table 6:** Dice, precision, recall and relative overlap (means and standard deviations computed over 10 rounds) are reported for RUSBoost, Adaboost, RF and FreeSurfer v.5.1 segmentations on DB2 MRI data set

	Dice	Precision	Recall	R.O.
*Left*				
RUSBoost	0.8670 $\pm$ 0.0305	0.8872 $\pm$ 0.0420	0.8598 $\pm$ 0.0477	0.7664 $\pm$ 0.0454
Adaboost	0.7392 $\pm$ 0.0329	0.7723 $\pm$ 0.0428	0.7140 $\pm$ 0.0598	0.5873 $\pm$ 0.0406
RF	0.8607 $\pm$ 0.0314	0.8801 $\pm$ 0.0422	0.8356 $\pm$ 0.0521	0.7568 $\pm$ 0.0460
FreeSurfer	0.7130 $\pm$ 0.0329	0.7390 $\pm$ 0.0444	0.6930 $\pm$ 0.0553	0.5550 $\pm$ 0.0400
*Right*				
RUSBoost	0.8594 $\pm$ 0.0725	0.8772 $\pm$ 0.0546	0.8501 $\pm$ 0.0940	0.7591 $\pm$ 0.0901
Adaboost	0.6938 $\pm$ 0.0632	0.7388 $\pm$ 0.0693	0.6600 $\pm$ 0.0837	0.5344 $\pm$ 0.0681
RF	0.8485 $\pm$ 0.0755	0.8844 $\pm$ 0.0520	0.8191 $\pm$ 0.0981	0.7428 $\pm$ 0.0927
FreeSurfer	0.7200 $\pm$ 0.0375	0.7540 $\pm$ 0.0478	0.6910 $\pm$ 0.0531	0.5630 $\pm$ 0.0475

**Fig. 3:**
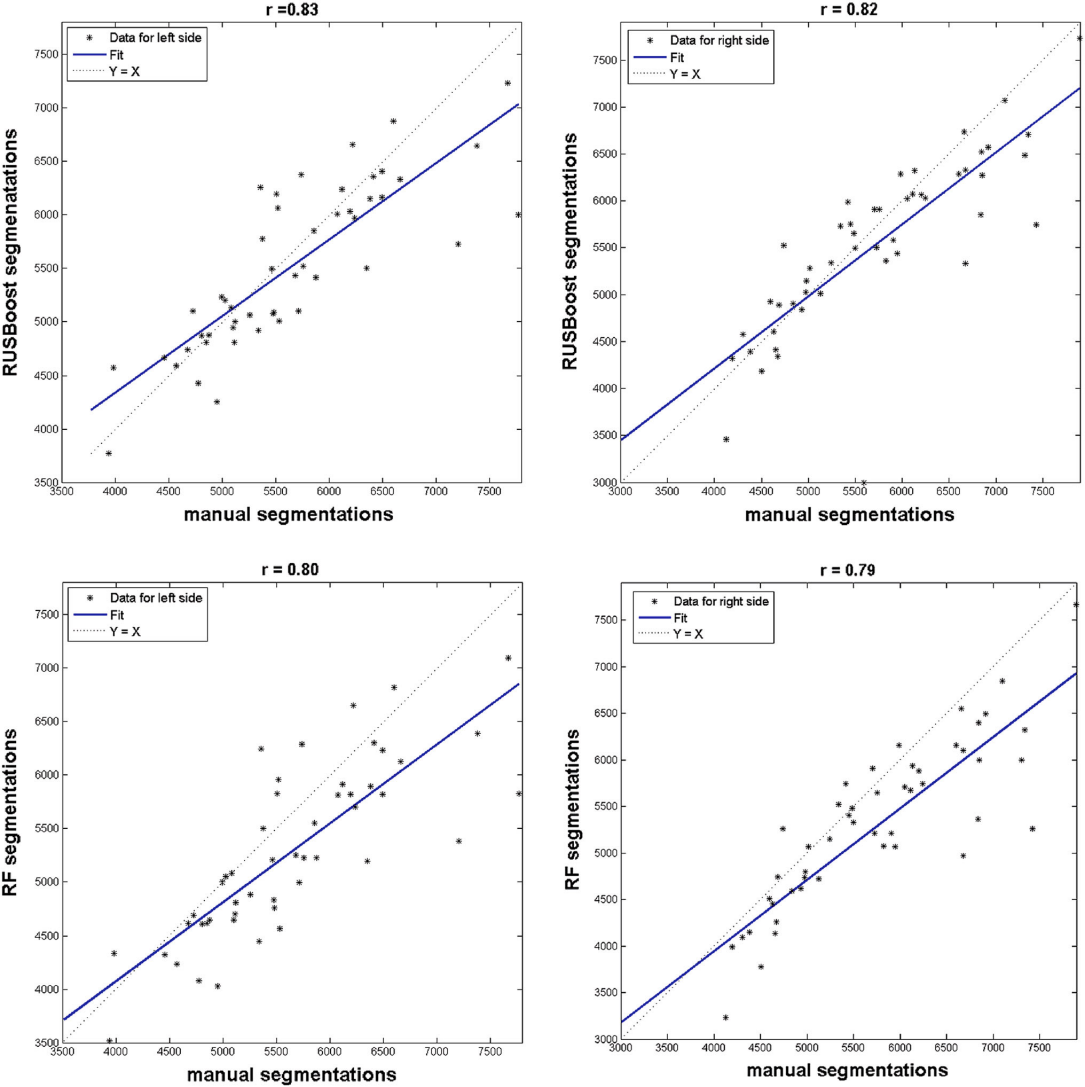
Scatter plots of the hippocampal volumes computed by the manual (target) and automated (output) segmentations on left and right brain hemispheres. The automated tracing was performed by RUSBoost and RF algorithms. The linear regressions of target relative to output are plotted and the Pearson regression coefficients (*r*) between manual and automated volumes are shown

Figure 3 shows the scatter plots and linear fits of the hippocampal volumes obtained using the manual tracing and the two best automated segmentations measured by RUSBoost and RF. The hippocampal volumes measured by RUSBoost showed the best agreement with the manually segmented volumes with a Pearson correlation coefficient *r*$= 0.83$ (0.82) for left (right) side, statistically significant (*p* value $= 1 \times 10^{-13}$). We also performed a paired two-sided sign test of the null hypothesis that the difference between volumes obtained by automated and manual segmentations comes from a continuous distribution with zero median, against the alternative that the distribution does not have zero median. For RF segmentation, the results of the sign test indicated a rejection of the null hypothesis at the $5\,\%$ significance level, with* p* value $= 6.17 \times 10^{-5}$ for the left and *p* value $= 3.63 \times 10^{-7}$ for the right side. Hence the hypothesis that the difference between volumes measured by RF segmentation and volumes obtained by manual tracing comes from a continuous distribution with zero median was rejected. For RUSBoost segmentation, at the $5\,\%$ significant level the test fails to reject the null hypothesis, therefore we cannot reject that the difference between volumes measured by RUSBoost segmentation and volumes obtained by manual tracing comes from a continuous distribution with zero median, with *p* value $= 0.152$ for the left and *p* value $= 0.253$. Overall, these are very encouraging results for a possible diagnostic use of this method and represent further evidence of the great potential of the proposed strategy for automated tissue segmentation.

## Conclusions

The use of automated techniques for image segmentation and analysis is gradually overtaking manual methods, particularly when applied to highly prevalent conditions, such as AD [11] and temporal lobe epilepsy [37], both disorders in which the hippocampus plays a pivotal role in the pathogenesis of the illness.

In this paper, we propose a novel strategy for automated segmentation of the hippocampal region based on the classifier RUSBoost, which produced excellent results when compared with other two learning methods, Adaboost and RF, and the publicly available package, FreeSurfer. For all experiments described in this paper, the classifiers were learning generalizable methods. RUSBoost gave the best results in terms of evaluation metrics; RF was the next best, suggesting that RUSBoost and RF may perform much better than both Adaboost and FreeSurfer.

RUSBoost proved to be the most accurate, with high sensitivity and precision. Moreover, the hippocampal volumes measured by RUSBoost showed the highest, statistically significant correlation with manually segmented volumes.

Some of the differences in the results obtained using different segmentation methods may be ascribed to the fact that the tools have been trained and tuned on different databases. Differences in image quality, manual segmentation protocol, clinical status and demographics have been described as possible causes of discrepancy [38]. An advantage of using machine learning algorithms for segmentation is the opportunity of using very large training data sets, shared by the scientific community. This is exemplified by the efforts of the EADC-ADNI working group to develop a standard harmonized protocol for the manual segmentation [8, 31, 32] (http://www.hippocampal-protocol.net) employed in our analysis.

This study was performed blindly to subject status. In terms of further developments, future efforts will be devoted to the application of these techniques to multiple data sets and other illness models. This approach could be extended to the study of other anatomical structures that have proved rather elusive to accurate segmentation, such as the thalamus or the putamen, both complex deep gray matter structures.

Overall, the results obtained with automated segmentation are very promising and a better understanding of the characteristics of the main machine learning methods is necessary for future applications combining multiple biomarkers and different illness sub-types.

## Evaluation metrics

A number of standard metrics described below were used to compare the performances of the four segmentation algorithms. Two binary vectors A and B are considered. A contains the voxel labels as identified by manual tracing and B contains the voxel labels predicted using a supervised learning algorithm. The voxels that the classifier correctly identifies as belonging to the hippocampus represent the true positives (TP) (i.e. intersection of A and B), the voxels correctly identified as background the true negatives (TN); the voxels wrongly identified as belonging to the hippocampus are the false positives (FP), and, finally, the voxels wrongly identified as background are the false negatives (FN).

Dice’s coefficient, precision, recall and relative overlap are defined as follows:

2 $$\begin{aligned}&\text {Dice} = \frac{2 TP}{(FP + TP) + (FN + TP)}\end{aligned}$$

3 $$\begin{aligned}&\text {Precision} = \frac{TP}{TP + FP}\end{aligned}$$

4 $$\begin{aligned}&\text {Recall} = \frac{TP}{TP + FN}\end{aligned}$$

5 $$\begin{aligned}&\text {R.O.} = \frac{TP}{FP + FN + TP} \end{aligned}$$

The Dice’s coefficient is an agreement measure over two sets of measures, A and B, defined as two times the ratio of the intersection of the two sets (i.e. TP) on the sum of A and B. Precision is defined by the ratio of the number of correct positive predictions on the number of total positive predictions. Recall measures the proportion of actual positives correctly identified from the number of all the actual positive examples. Relative overlap (R.O.) measures the similarity between two sets of measures as the size of the intersection divided by the size of the union of the sets.

## Classifiers

### Adaboost

Adaboost is a meta-algorithm that sequentially selects weak classifiers, and weighs each of them based on their error. A weak classifier is a classifier that performs better than pure chance. The algorithm assigns to each example the weight $D_1(i) = \frac{1}{m}$. Then, in each round $t = 1, 2, \ldots , T$, the following steps are performed:

1. Training of a weak learner $h_t: X \rightarrow \{-1, +1\}$ using the distribution $D_t$.
2. Calculation of the error $e_t = \sum _{i:h_t( x_ i)\ne y_i} D_t(i)$.
3. Setting of $\alpha _t = \frac{1}{2}\text {ln}\left(\frac{1-e_{\it {t}}}{e_{\it {t}}}\right)$, which measures the importance assigned to $h_t$. If $e_t \le \frac{1}{2}$ then $\alpha _t \ge 0$.
4. Setting of $D_{t+1}(i) = \frac{D_t(i)}{Z_t}e^{-\alpha _t y_i h_t({\mathbf x} _i)}$, where $Z_t = 2\sqrt{e_t(1-e_t)}$ is a normalization factor. $D_t(i)$ measures the importance assigned to the example $x_i$ at the iteration *t*.

The output of the strong classifier on a new example $\mathbf x$ is:

6 $$\begin{aligned} y = \text {sign}(f(\mathbf x)) = \text {sign}\left(\sum _{\it{t}=1}^{\it{T}} \alpha _t h_t(\mathbf x )\right). \end{aligned}$$

The algorithm tends to concentrate on hard examples, i.e. after selecting an optimal classifier $h_{t}$ for the distribution $D_{t}$, the examples $x_{i}$, that were identified correctly by the classifier $h_{t}$, are given lower weight, and those that were identified incorrectly by $h_{t}$ are given higher weight. Therefore, when the algorithm is testing the classifiers on the distribution $D_{t+1}$, it will select a classifier that better identifies those examples that the previous classifier missed.

The final hypothesis *y* is a weighted majority vote of the *T* weak hypothesis where $\alpha _t$ is the weight to $h_t$, that is the weighted mean of the *T* weak classification on $\mathbf x$.

### Random forest

Random Forest uses multiple binary decision trees. Each of the classification trees is built using a sample of the training data, and at each node a randomly chosen set of variables is considered for the best split.

For $b = 1, 2, \ldots, B$ the RF algorithms can be briefly described as follows.

1. A bootstrap sample **Z*** of size *n* is drawn from the training set.
2. A random forest tree $T_b$ is grown from the bootstrapped data, by recursively repeating the following steps for each terminal node of the tree, until the minimum node size, $n_{min}$, is reached:
  - Selection of *q* variables at random from the *d* variables;
  - Choice of the best variable/split-point among *q* (internal feature selection);
  - Splitting of the node into two daughter nodes.
3. Output of the ensemble of trees $\{T_b\}_1^B$.

Given a new point $\mathbf x$, let $\tilde{C}_b(\mathbf x )$ be the class prediction of the $b-$th random forest tree, the prediction of RF on this new sample is given by

$$\begin{aligned} y = \text{majority vote } \{{\tilde{C}}_b({\mathbf x})\}_1^B \end{aligned}$$

In the experiments here described, $q = \sqrt{d}$ and the minimum node size was 1.

## FreeSurfer

Cortical reconstruction and volumetric segmentation were performed with the FreeSurfer image analysis suite, which is documented and freely available for download online.^1^ The technical details of these procedures are described in prior publications [15, 39–49]. Briefly, this processing includes motion correction and averaging [50] of multiple volumetric T1 weighted images (when more than one is available), removal of non-brain tissue using a hybrid watershed/surface deformation procedure [48], automated Talairach transformation, segmentation of the subcortical white matter and deep gray matter volumetric structures (including hippocampus, amygdala, caudate, putamen, ventricles) [15, 42] intensity normalization [51], tessellation of the gray matter white matter boundary, automated topology correction [41, 52], and surface deformation following intensity gradients to optimally place the gray/white and gray/cerebrospinal fluid borders at the location where the greatest shift in intensity defines the transition to the other tissue class [39, 40, 49]. Once the cortical models are complete, a number of deformable procedures can be performed for in further data processing and analysis including surface inflation [39], registration to a spherical atlas which utilized individual cortical folding patterns to match cortical geometry across subjects [44], parcellation of the cerebral cortex into units based on gyral and sulcal structure [45, 53], and creation of a variety of surface-based data including maps of curvature and sulcal depth. This method uses both intensity and continuity information from the entire three-dimensional MR volume in segmentation and deformation procedures to produce representations of cortical thickness, calculated as the closest distance from the gray/white boundary to the gray/CSF boundary at each vertex on the tessellated surface [40]. The maps are created using spatial intensity gradients across tissue classes and are therefore not simply reliant on absolute signal intensity. The maps produced are not restricted to the voxel resolution of the original data thus are capable of detecting submillimeter differences between groups. Procedures for the measurement of cortical thickness have been validated against histological analysis [54] and manual measurements [55, 56]. Freesurfer morphometric procedures have been demonstrated to show good test-retest reliability across scanner manufacturers and across field strengths [46, 57].

### Example text for longitudinal processing

To extract reliable volume and thickness estimates, images where automatically processed with the longitudinal stream in FreeSurfer [57]. Specifically an unbiased within-subject template space and image [58] is created using robust, inverse consistent registration [50]. Several processing steps, such as skull stripping, Talairach transforms, atlas registration as well as spherical surface maps and parcellations are then initialized with common information from the within-subject template, significantly increasing reliability and statistical power [57].
